# Identification of circRNA-associated ceRNA networks in peripheral blood mononuclear cells as potential biomarkers for chronic obstructive pulmonary disease

**DOI:** 10.1042/BSR20230005

**Published:** 2023-10-31

**Authors:** Shan Zhong, Chengshui Chen, Li Yang, Meiling Jin, Yiming Zeng, Gang-Ming Zou, Qingying Zhang, Yun Wang

**Affiliations:** 1 College of Life Sciences and Oceanography, Shenzhen University, Shenzhen, Guangdong 518055, PR China; 2 Department of Preventive Medicine, Shantou University Medical College, Shantou, Guangdong 515041, PR China; 3 Institute of Precision Medicine, Peking University Shenzhen Hospital, Shenzhen, Guangdong 518036, PR China; 4 Department of Respiratory Medicine, First Affiliated Hospital of Wenzhou Medical University, Wenzhou, Zhejiang 325000, PR China; 5 Department of Respiratory Medicine, Zhongshan Affiliated Hospital of Fudan University, Shanghai 200030, PR China; 6 Department of Respiratory Medicine, Second Affiliated Hospital of Fujian Medical University, Quanzhou, Fujian 362000, PR China; 7 School of Nursing and Dental Health. University of Hawaii at Manoa, 2528 McCarthy Mall, Webster Hall. Honolulu, HI 96822, USA

**Keywords:** biomarkers, ceRNA, circRNAs, COPD, pathogenesis

## Abstract

Chronic obstructive pulmonary disease (COPD), which is a common respiratory disorder with high morbidity and mortality globally, has a complex pathogenesis that is not fully understood. Some circular RNAs (circRNAs) have been recognized to serve as miRNA sponges for regulating target RNA transcripts during the processes of human diseases. In the present study, we aimed to investigate novel circRNA-associated biomarkers for COPD, 245 differentially expressed circRNAs were identified, including 111 up-regulated and 134 down-regulated circRNAs. These candidate circRNAs were enriched in inflammation-associated pathways (such as mTOR, B-cell receptor, and NF-κB signaling pathways) via Gene Ontology and Kyoto Encyclopedia of Genes and Genomes enrichment analyses. A combination of two circRNAs (up-regulated hsa_circ_0067209 and down-regulated hsa_circ_0000673) demonstrated good diagnostic value (area under the receiver operating characteristic curve [AUC] = 0.866) for COPD by receiver operating characteristic curve (ROC) analysis and qRT-PCR validation. Subsequently, hsa-miR-8082 and hsa-miR-1248 were identified as targets for hsa_circ_0067209 and hsa_circ_0000673, respectively, via bioinformatics analysis and a dual-luciferase reporter assay, and the combination of these two miRNAs displayed better diagnosis potential for COPD (AUC = 0.967) than each other. Evaluation of COPD-related mRNA profiles revealed that the up-regulated genes *ABR* and *TRPM6* were predicted downstream targets for hsa_circ_0067209/hsa-miR-8082, whereas the down-regulated gene *RORC* was a predicted downstream target for hsa_circ_0000673/hsa-miR-1248. In summary, hsa_circ_0067209 and hsa_circ_0000673 have potential as novel diagnostic biomarkers of COPD. In addition, competing endogenous RNA networks of hsa_circ_0067209/hsa-miR-8082/ABR/TRPM6 and hsa_circ_0000673/hsa-miR-1248/RORC may play critical regulation roles for COPD pathogenesis.

## Background

Chronic obstructive pulmonary disease (COPD) is a heterogeneous respiratory disorder with chronic inflammation and incomplete reversible airflow obstruction, which leads to higher morbidity and mortality and poses a heavy social and economic burden globally [1–3]. In 2018, a China Pulmonary Health (CPH) study by Wang et al. [4] reported that the overall prevalence of spirometry-defined COPD in individuals >40 years of age had increased by 13.7%, amounting to 99.9 million patients with COPD. A major etiological factor of COPD is long-term exposure to noxious particles and/or gas [5]. However, the detailed pathophysiological mechanism of COPD is not well understood.

Non-coding RNA (ncRNA) is classified into two subtypes: linear ncRNA and circular RNA (circRNA). Although circRNA possesses the primary structural characteristics of linear ncRNA, it contains covalently closed loops without a 5′ cap and a 3′ poly (A) tail, resulting in a configuration that is more conserved and stable than linear ncRNAs in the cytoplasm of eukaryotic cells [6]. In the past decade, increasing evidence has shown that circRNAs play important roles in the development of various diseases by regulating the expression and function of mRNAs, miRNAs, and proteins [7–10].

**Figure 1:**
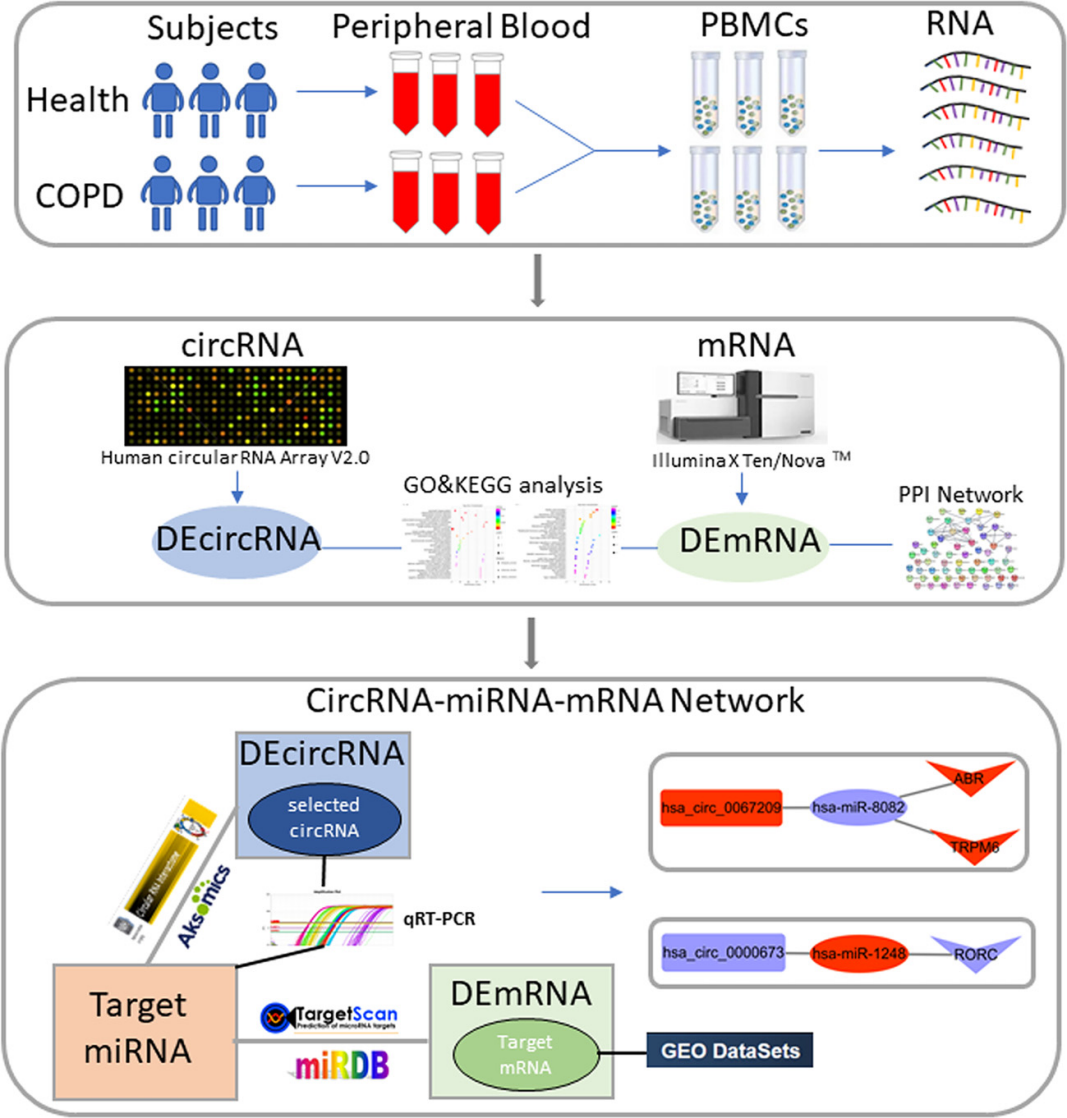
The workflow of the present study DEcircRNA, differentially expressed circRNA; DEmRNA, differentially expressed mRNA.

Recently, the regulatory roles of circRNA in gene transcription and alternative splicing have been elucidated, indicating that some circRNAs may serve as miRNA sponges or may combine with RNA-binding proteins to affect RNA/protein synthesis and degradation [11]. For example, co-expression of cirs-7/CDR1as and miR-7 in nerve tissues *in vivo* was closely associated with critical regulatory pathways in neurological disorders [12,13]. Moreover, circRNAs have been recognized as regulators in the processes of cancer. For example, circRNA_LARP4 acts as a sponge for miR-424-5p to regulate LATS1 expression and inhibits cell proliferation in invasive gastric cancer [14]. In addition, circMTO1 could be directly bound to matched microRNA in hepatocellular carcinoma, thereby behaving as a prognostic biomarker [15]. Furthermore, aberrant levels of circRNA expression have been reported in a various respiratory diseases, such as lung cancer [16,17], pulmonary arterial hypertension [18], asthma [19], and idiopathic pulmonary fibrosis [20]. Zeng and coworkers [21] showed that cigarette smoke extract (CSE) could stimulate primary human epithelial cells of small airways in a cellular model of COPD, suggesting that some circRNAs may perform key roles through specific circRNA-mediated competing endogenous RNA (ceRNA) networks in COPD. However, there were very few circRNA studies on COPD development. Consequently, in the present study, we aimed to screen out COPD-related biomarkers from peripheral blood mononuclear cells (PBMCs) by a comparison between COPD patients and healthy controls, and then explore potential roles of circRNA-miRNA networks and associated gene effectors in the COPD process. The workflow of the present study is shown in Figure 1.

## Methods

### Clinical samples

Peripheral blood samples of all participants in the present study were collected at the First Affiliated Hospital of Wenzhou Medical University (Wenzhou, China) from December 2017 to December 2019. The study was carried out in accordance with the World Medical Association Declaration of Helsinki, approved by the Human Medical Ethics Committee of the First Affiliated Hospital of Wenzhou Medical University (Approval no. 2016131), and written informed consent was provided by each participant. The participants were 40 to 80 years old and were grouped based on their clinical respiratory symptoms. Participants with a history of respiratory symptoms (e.g., chronic cough, wheezing, and/or expectoration) and a ratio of forced expiratory volume in 1st second (FEV1) to forced vital capacity (FVC) less than 70% after inhalation of albuterol were defined as COPD. Exclusion criteria included a history of malignancy, cardiovascular diseases, Alzheimer’s disease, autoimmune disease, and other respiratory diseases (such as bronchiectasis, bronchial asthma pulmonary fibrosis, and active tuberculosis, etc.). The basic clinical characteristics of all subjects are provided in Tables 1-3.

**Table 1:** Clinical information of participants used in circRNA microarray analysis

Characteristic	Control (*n*=9)	COPD (*n*=9)
Gender (male/female)	9/0	9/0
Age (years)	55.000 ± 4.583	59.333 ± 6.834
BMI (kg/m^2^)	24.873 ± 3.061	22.076 ± 3.833
Current/ex-smokers	7/9	6/9
**Pulmonary functions**		
FVC (L)	2.939 ± 0.482	1.781 ± 0.776
FEV1 (L)	2.741 ± 0.363	0.927 ± 0.408
FEV1/FVC%	92.389 ± 7.088	35.511 ± 16.894
FEV1% predicted	98.676 ± 9.788	49.701 ± 16.503

**Table 2:** Clinical information of participants used for circRNA validation

Characteristic	Control (*n*=36)	COPD (*n*=36)
Gender (male/female)	25/11	33/3
Age (years)	56.361 ± 9.206	66.802 ± 6.803
BMI (kg/m^2^)	24.123 ± 3.165	21.406 ± 3.572
Current/ex-smokers	17/23	10/30
**Pulmonary functions**		
FVC (L)	3.064± 0.632	2.330 ± 0.982
FEV1 (L)	2.582 ± 0.531	1.458± 0.846
FEV1/FVC%	94.589± 16.159	53.406 ± 29.509
FEV1% predicted	85.506 ± 7.514	59.080 ± 15.710

**Table 3:** Clinical information of patients used for miRNA validation

Characteristic	Control (*n*=24)	COPD (*n*=24)
Gender (male/female)	16/6	20/4
Age (years)	55.118 ± 10.006	63.000 ± 9.393
BMI (kg/m^2^)	24.384 ± 2.839	20.331 ± 2.828
Current/ex-smokers	11/16	10/19
**Pulmonary functions**		
FVC (L)	3.395 ± 0.658	2.312 ± 1.024
FEV1 (L)	2.802 ± 0.542	1.462 ± 0.821
FEV1/FVC%	93.129 ± 12.264	51.339 ± 24.893
FEV1% predicted	85.488 ± 9.571	55.639 ± 16.024

### Isolation of PBMCs and extraction of RNA

Peripheral blood samples from COPD patients and healthy controls were collected into EDTA-anticoagulated vacutainer tubes. PBMCs were isolated from 10 ml of blood sample per case by density gradient centrifugation using human lymphocyte separation medium (Solarbio Life Sciences, China) and were immediately stored at −80°C until the assay. Total RNA was extracted from the PBMCs of each case with the M5 HiPer Universal Plus RNA Mini Kit (Mei5 Biotechnology, China), according to the kit instructions. The concentration of total RNAs were measured with the Nanodrop ND-2000 spectrophotometer.

### Microarray analysis

Total RNAs were initially extracted from PBMC samples of nine COPD patients and nine healthy subjects. Three cases with equal quality of RNAs per group were pooled into one testing sample. Subsequently, three pooled samples per group were applied to microarray testing. Approximately 3 μg of RNA per sample was digested with 3 U/μg of RNase R (Epicentre, U.S.A.) for 20 min at 37°C to purify the circRNAs. The enriched circRNAs were then reversed transcribed into cRNA utilizing fluorescent reagents with random primers, and were hybridized onto the Arraystar Human circRNA Array V2 (8 × 15K, Arraystar, U.S.A.). The circRNA expression profiles were analyzed with the Arraystar program and the limma package of R software. CircRNAs with |fold change| >2 and adjusted *P-*value <0.05 were considered significantly differentially expressed. The disease-related circRNA candidates were screened out based on the back-splice junction of the special structure of RNA and were confirmed by Sanger sequencing of amplification products (Sangon Biotech, China).

### RNA sequencing analysis

Specific libraries were constructed from three pooled-COPD RNA samples and three pooled-control RNA samples after removal of rRNAs. Subsequently, paired-end sequencing, generating 150-bp reads, was performed on the Illumina X Ten/Nova™ platform. The differentially expressed genes (DEGs) were obtained from a comparison between COPD and control groups using the DESeq2 package of R software (Version 1.20.0, http://www.bioconductor.org/packages/release/bioc/html/DESeq2.html). Genes with cutoff values of |fold change| >2 and adjusted *P-*value <0.05 were identified as DEGs.

### Luciferase activity assay

Wild-type (WT) and mutated (MUT) hsa_circ_0067209 sequences were co-transfected with NC-mimic or hsa-miR-8082 mimic into HEK293T cells with the psiCHECK-2 Luciferase Reporter Vectors (Promega, U.S.A.). In addition, WT and MUT hsa_circ_0000673 sequences were co-transfected with NC-mimic or hsa-miR-1248 into HEK293T cells using the same technique. Treated cells were lysed after incubating for 48 h, and luciferase activity was measured by the Dual-Luciferase Reporter Assay System (Promega), according to the manufacturer’s protocol.

### Quantitative real-time PCR (qRT-PCR)

Specific paired primers for qRT-PCR were designed and synthesized by Sangon Biotech (Supplementary Table S1). Universal reverse primers and U6 primers were provided by the Mir-X miRNA First-Strand Synthesis Kit (TaKaRa, Japan), and used for miRNA expression analysis. cDNAs were synthesized from total RNAs with a cDNA synthesis kit (TaKaRa) or the Mir-X miRNA First-Strand Synthesis Kit. Subsequently, qRT-PCR amplification was performed via SYBR Green PCR Premix Ex TaqTM II reagents (TaKaRa) with the QuantStudio 6 FlexI real-time system (Applied Biosystems, U.S.A.) following the protocol of this product. The levels of targeted genes were determined with the 2^(−ΔΔCt)^ method in comparison with endogenous controls (GAPDH or U6).

### Gene Expression Omnibus (GEO) datasets for mRNA validation

To validate the expression levels of target mRNAs in the network, two microarray datasets associated with COPD (GSE57148 and GSE54837) were downloaded from the GEO public data repository (http://www.ncbi.nlm.nih.gov/geo). The GSE57148 dataset contains 189 samples of lung tissues (including 91 cases of normal individual and 98 cases of patients with COPD. The GSE54837 dataset contains 226 blood samples from patients with COPD in different stages (Stage 1: *n*=90, Stage 2: *n*=68, Stage 3: *n*=55, Stage 4: *n*=13).

### Biological function and signal pathway enrichment analyses

An online tool (https://cloud.oebiotech.cn) was used for functional annotation of candidate genes. Gene Ontology (GO) analysis was performed to enrich biological functions of screened out DEGs, including molecular function, cellular components, and biological processes. Kyoto Encyclopedia of Genes and Genomes (KEGG) analysis was applied to enrich signal pathways of disease-related DEGs.

### Protein–protein interaction (PPI) network analysis

STRING version 11.0b (https://cn.string-db.org/), Cytoscape version 3.5 (https://cytoscape.org/), and the MCODE app were applied for establishing the PPI networks of mRNA. Parameter sets were Network Scoring (Include Loops = false, Degree Cutoff = 2) and Cluster Finding (Node Score Cutoff = 0.2, Haircut = true, Fluff = false, K-Core = 2, Max. Depth from Seed = 100).

### Construction of circRNA-miRNA-mRNA networks

The Circular RNA Interactome online tool (https://circinteractome.irp.nia.nih.gov/), miRadna (http://www.microrna.org/), and TargetScan (http://www.targetscan.org/) were used to predict targeted miRNAs of candidate circRNAs in the present study. The binding sites of circRNA-miRNA were visualized via a platform from the online website (https://cloud.oebiotech.cn/). The interactions of miRNA-mRNA were predicted via miRDB (http://mirdb.org/) and TargetScan software. Next, special circRNA-miRNA and miRNA-mRNA pairs were combined to construct circRNA-miRNA-mRNA networks that were visualized by Cytoscape version 3.5 (https://cytoscape.org/).

### Statistical analysis

GraphPad Prism 6.0 (GraphPad Software Inc., San Diego, CA, U.S.A.) and SPSS V21.0 (NY: IBM Corp, Armonk, U.S.A.) were used as statistical tools. Student’s *t*-test and Mann–Whitney *U*-test were applied to measure the differences of gene expression levels between COPD and control groups after normalizing data. Receiver operating characteristic (ROC) curve analysis was performed to evaluate the power of candidate genes. The correlation of gene expression and clinical characteristics in the cohort study was calculated using Spearman rank correlation. *P*<0.05 was considered statistically significant.

## Results

### Screening out COPD-related circRNAs by circRNA microarray

**Figure 2:**
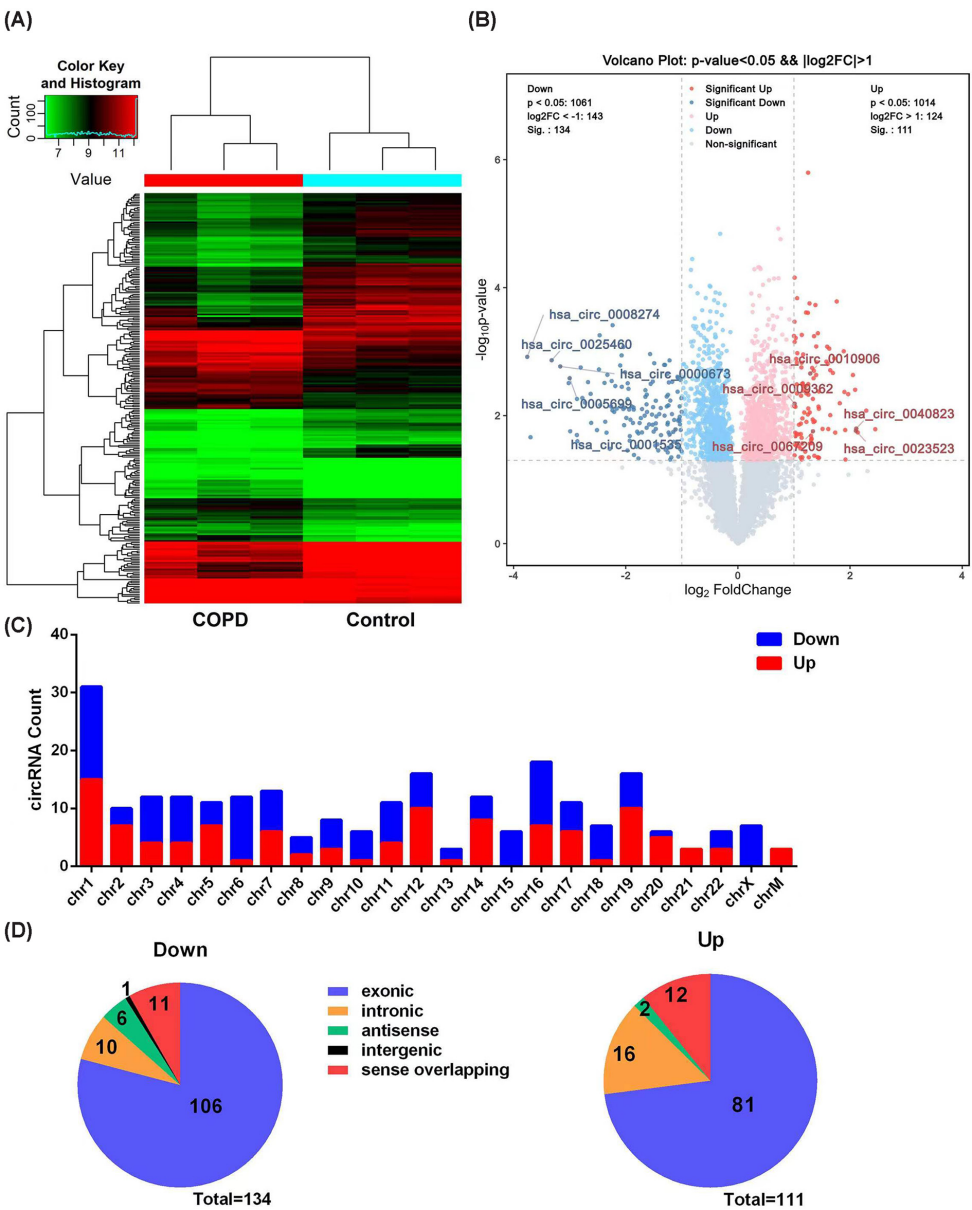
Identification of differentially expressed circRNAs in COPD by microarray analysis (**A**) Heatmaps of differentially expressed circRNAs between COPD patients and healthy controls. ‘Red’ indicates higher relative expression, and ‘green’ indicates lower relative expression. (**B**) Volcano map of differentially expressed circRNAs from COPD and control groups. The red and blue points in the plot represent the up-regulated and down-regulated circRNAs, respectively. The circRNAs with fold change >2 and *P*<0.05 were considered to be differential genes, and ten circRNAs investigated in the present study are annotated in the volcano plot. (**C**) The distribution of differentially expressed circRNAs located in the chromosomes. (**D**) Types of differentially expressed circRNAs.

To screen for COPD-related circRNAs, the circRNA expression levels in PBMCs from COPD patients and healthy controls (three pooled cases per group) were detected with a human circRNA microarray. Comparison and bioinformatics analyses were subsequently conducted to select disease-related differentially expressed circRNAs from the COPD group versus the control group. Hierarchical clustering results (Figure 2A) showed that there were various differentially expressed circRNAs between the COPD patients and healthy controls. Volcano plots based on the specific threshold of |fold change| >2 and *P*<0.05 were used to identify differentially expressed circRNAs between the COPD patients and controls (Figure 2B). A total of 245 differentially expressed circRNAs were found in COPD patients, compared with healthy controls, including 111 up-regulated and 134 down-regulated circRNAs. The top 20 circRNAs are listed in Table 4, according to the values of expressive fold change. Analysis of the chromosome localization and classification of the circRNA candidates in COPD (Figure 2C) indicated that the differentially expressed circRNAs were widely distributed in all chromosomes but were primarily derived from chromosomes 1, 12, 16, and 19. Moreover, approximately three-quarters of the identified candidates were rooted in exons (Figure 2D).

**Table 4:** Top 20 differentially expressed circRNAs related to COPD

circRNA ID	Circbase ID	Fold change	*P*-value	Gene symbol	circRNA_type
**Upregulated circRNAs**					
hsa_circRNA_089763	hsa_circ_0089763	5.465	0.016	*JA760600*	Exonic
hsa_circRNA_406587	-	4.890	0.008	*TRIO*	Intronic
hsa_circRNA_001678	hsa_circ_0000517	4.399	0.018	*RPPH1*	Sense overlapping
hsa_circRNA_101903	hsa_circ_0040823	4.319	0.016	*BANP*	Exonic
hsa_circRNA_023523	hsa_circ_0023523	4.298	0.017	*UCP2*	Exonic
hsa_circRNA_009054	hsa_circ_0009054	4.262	0.004	*MCC*	Exonic
hsa_circRNA_051238	hsa_circ_0051238	4.149	0.002	*ATP5SL*	Exonic
hsa_circRNA_001065	hsa_circ_0001065	3.912	0.005	*GYPC*	Antisense
hsa_circRNA_060102	hsa_circ_0060102	3.784	0.049	*ERGIC3*	Exonic
hsa_circRNA_040730	hsa_circ_0040730	3.770	0.017	*GSE1*	Exonic
**Downregulated circRNAs**					
hsa_circRNA_101287	hsa_circ_0008274	13.516	0.001	*UGGT2*	Exonic
hsa_circRNA_406083	-	12.981	0.022	*TASP1*	Intronic
hsa_circRNA_025460	hsa_circ_0025460	9.997	0.001	*YBX3*	Exonic
hsa_circRNA_101707	hsa_circ_0000673	8.991	0.002	*RSL1D1*	Exonic
hsa_circRNA_404837	-	8.093	0.003	*NUP98*	Intronic
hsa_circRNA_101744	hsa_circ_0005699	7.999	0.003	*C16orf62*	Exonic
hsa_circRNA_001655	hsa_circ_0001655	7.947	0.018	*-*	Intergenic
hsa_circRNA_050649	hsa_circ_0050649	7.317	0.020	*HSPB6*	Exonic
hsa_circRNA_100983	hsa_circ_0024766	7.311	0.005	*STT3A*	Exonic
hsa_circRNA_033628	hsa_circ_0033628	7.269	0.025	*CRIP1*	Exonic

### GO and KEGG enrichment analysis of COPD-related circRNAs

**Figure 3:**
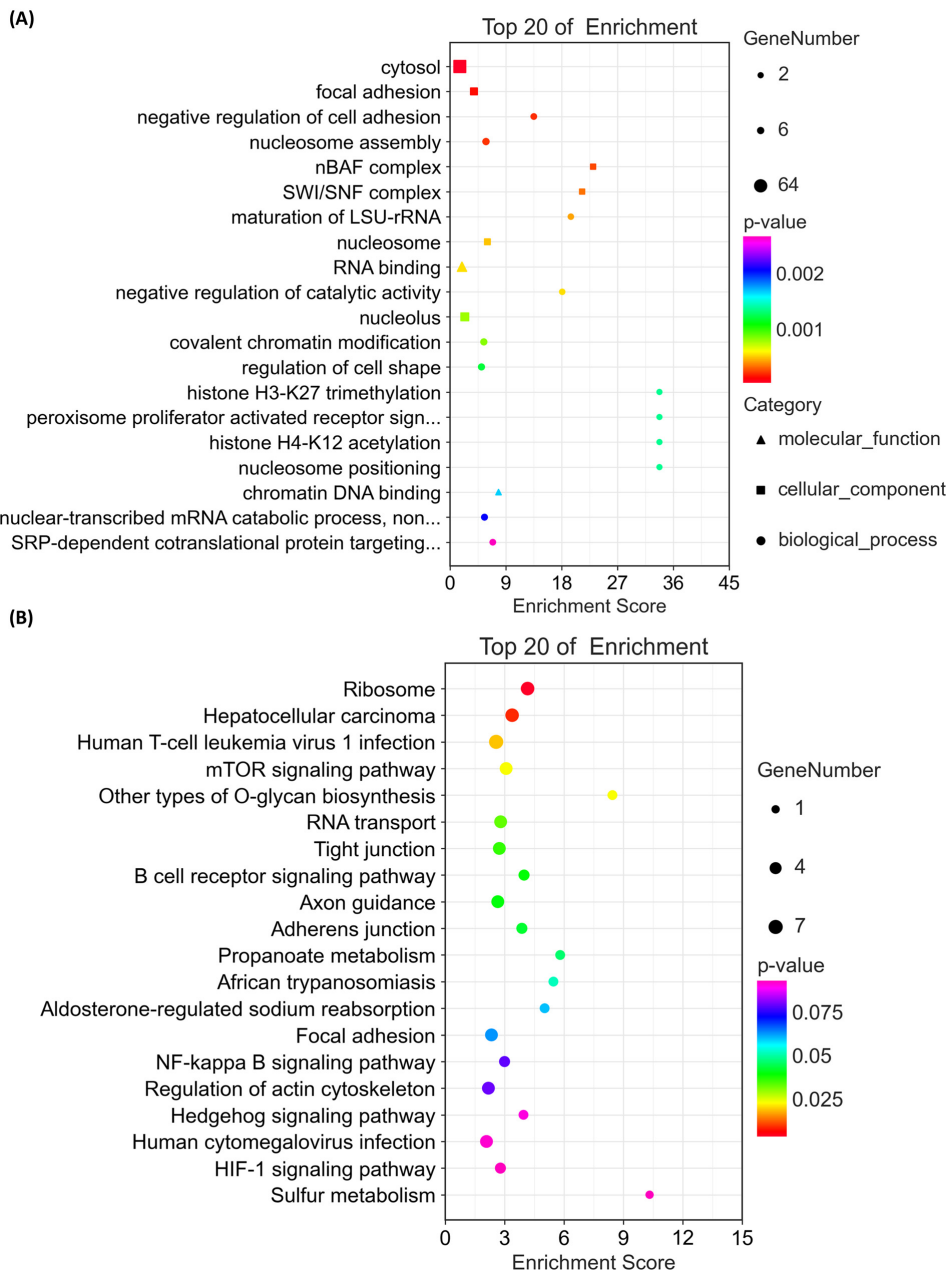
Enrichment analyses of differentially expressed circRNAs in COPD Differentially expressed circRNAs were subjected to (**A**) GO analysis and (**B**) KEGG pathway analysis.

GO analysis results are illustrated in Figure 3A. In molecular function, the COPD-related circRNAs were chiefly incorporated into RNA binding and chromatin DNA binding. In cellular component, the circRNAs were primarily included in the cytosol, focal adhesion, and nucleosome. In biological process, the candidate circRNAs were principally involved in the regulation of cell adhesion and nucleosome assembly. Concurrently, KEGG enrichment analysis of the COPD-related candidate circRNAs indicated that they were primarily enriched in genetic information processing consisting of ribosome and RNA transport, and in signal pathways such as the mTOR, B-cell receptor, and NF-κB signaling pathways (Figure 3B).

### Validation of candidate circRNAs in PBMCs of patients with COPD

**Figure 4:**
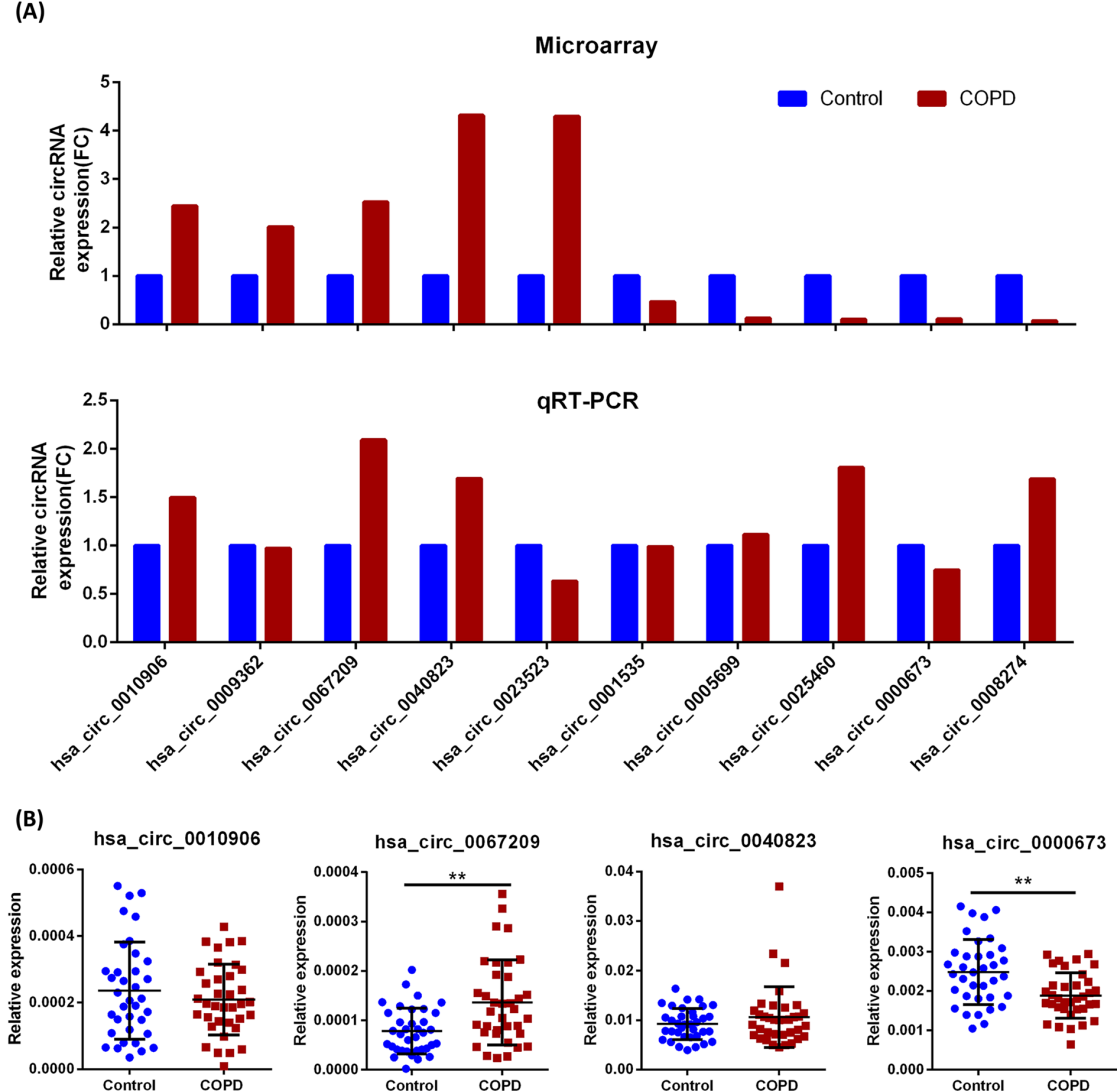
Expression validation of candidate circRNAs between COPD patients and controls (**A**) Preliminary comparison of microarray and qRT-PCR data for the relative expression levels of selected circRNAs. (**B**) Expressions of hsa_circ_0010906, hsa_circ_0067209, hsa_circ_0040823, and hsa_circ_0000673 were measured with 72 additional samples by qRT-PCR. Control: *n*=36, COPD: *n*=36. Relative expression levels were presented as 2^(−ΔCt)^, GAPDH was used as an internal reference; **P<0.01, *P<0.05.

Through expression abundance and specificity target sequencing (Supplementary Figure S1), ten differentially expressed circRNAs were selected for validation by qRT-PCR, including five up-regulated circRNAs (hsa_circ_0010906, hsa_circ_0009362, hsa_circ_0067209, hsa_circ_0040823, and hsa_circ_0023523) and five down-regulated circRNAs (hsa_circ_0001535, hsa_circ_0005699, hsa_circ_0025460, hsa_circ_0000673, and hsa_circ_0008274). The qRT-PCR results indicated that the expression levels of hsa_circ_0010906, hsa_circ_0067209, hsa_circ_0040823, and hsa_circ_0000673 had similar trends to those of the microarray analysis (Figure 4A). The expression levels of these four circRNAs were reconfirmed with new collected 72 samples for extentional testing. The results showed that hsa_circ_0067209 was significantly up-regulated and hsa_circ_0000673 was markedly down-regulated in COPD, compared with the controls (Figure 4B). The circRNA hsa_circ_0067209 was derived from the eukaryotic elongation factor selenocysteine-tRNA specific (EEFSEC) gene, which had a spliced sequence length of 470 bp and consisted of the head-to-tail splicing of exons 2, 3, and 4 (Supplementary Figure S2A). The circRNA hsa_circ_0000673 was derived from exon regions 4 and 5 within the ribosomal L1 domain-containing protein 1 (RSL1D1) gene locus, and the spliced mature sequence was 251 bp in length (Supplementary Figure S2B). The circular characteristics of hsa_circ_0067209 and hsa_circ_0000673 were verified after RNase R digestion, and both circRNAs exhibited more resistance to RNase R digestion compared with that of matched linear mRNAs (Supplementary Figure S2C).

### Diagnostic value evaluation for hsa_circ_0067209 and hsa_circ_0000673 in COPD

**Figure 5:**
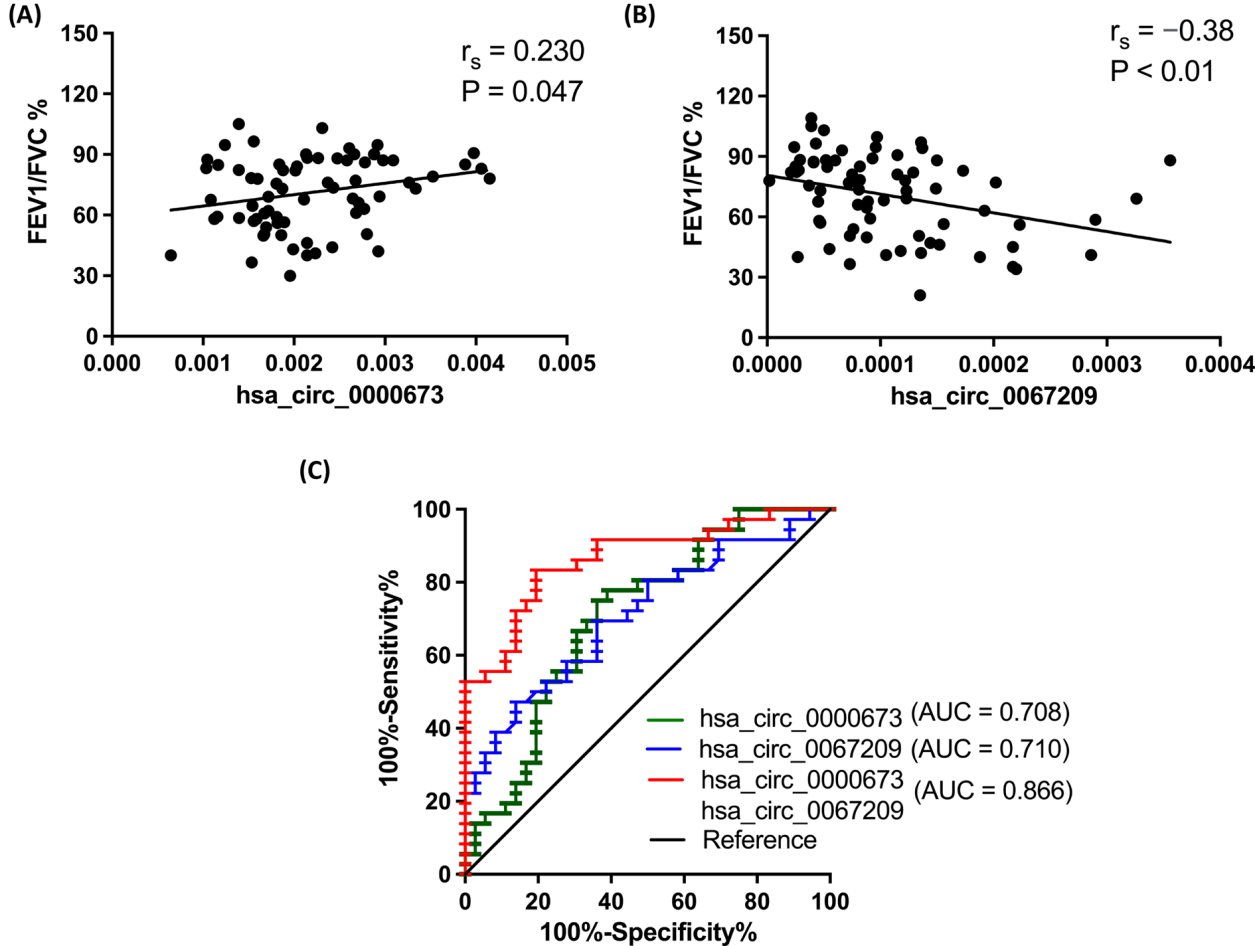
Correlation analysis of clinical characteristics and ROC curve analysis of hsa_circ_0000673 and hsa_circ_0067209 (**A,B**) Spearman correlation analysis for the link between the FEV1/FVC% of lung function and the expression of hsa_circ_0000673 or hsa_circ_0067209. (**C**) ROC curve analysis for hsa_circ_0000673, hsa_circ_0067209, and the combination of both circRNAs.

As a sensitive and reliable indicator, the lung function decrease (FEV1/FVC%≤70%) is the most important diagnosis index of COPD [1]. In the present study, the clinical characteristics of all subjects suggested that abnormal expression of hsa_circ_0000673 and hsa_circ_0067209 were significantly related to FEV1/FVC% (Figure 5A,B). Data from ROC curve analysis showed that the area under the ROC curve (AUC) values of hsa_circ_0067209 and hsa_circ_0000673 were 0.710 (95% confidence interval [CI]: 0.591–0.829) and 0.708 (95% CI: 0.587–0.828), respectively. Moreover, the AUC value of the two circRNAs in combination was 0.866 (95% CI: 0.782–0.950) (Figure 5C), suggesting that the combination provided better diagnostic value than the circRNAs individually.

### Verification of targeted miRNAs for hsa_circ_0067209 or hsa_circ_0000673

**Figure 6:**
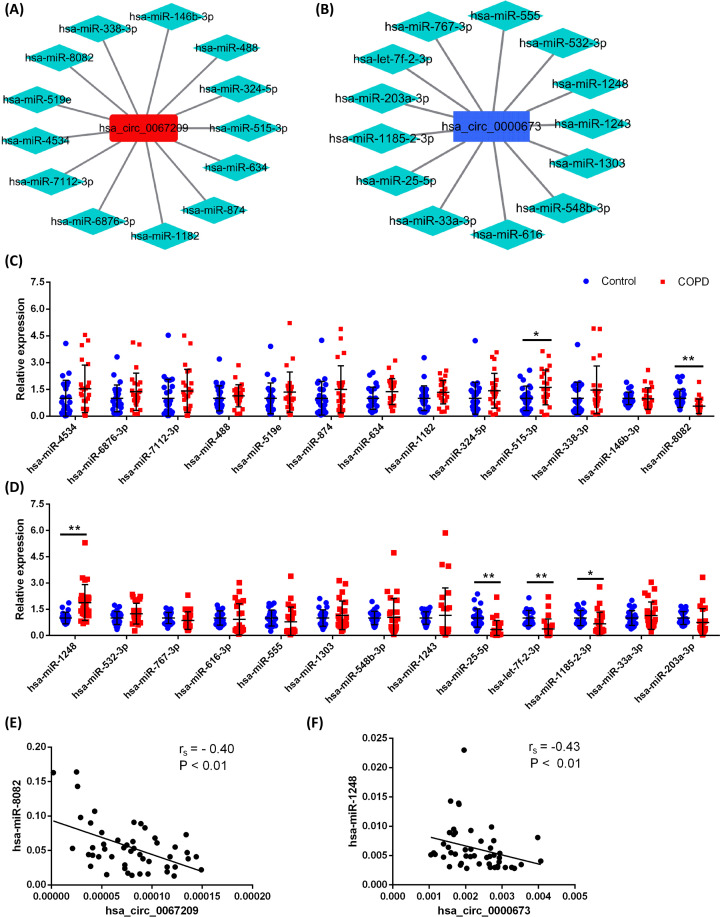
Prediction and expression validation of sponging miRNAs of hsa_circ_0067209 and hsa_circ_0000673 (**A,B**) The interactive network of circRNA-miRNA was constructed for hsa_circ_0067209 and hsa_circ_0000673. (**C,D**) Expression levels of predicted candidate miRNAs for hsa_circ_0067209 or hsa_circ_0000673 by qRT-PCR. Control: *n*=24, COPD: *n*=24. Relative expression was presented as 2^(−ΔCt)^, and U6 was used as an internal reference. (**E**) Correlation analysis of hsa_circ_0067209 and hsa-miR-8082. (**F**) Correlation analysis hsa_circ_0000673 and hsa-miR-1248; **P<0.01, *P<0.05.

CircRNAs are recognized to have negative regulatory capability for miRNA expression, based on the hypothesis of ceRNA [11–13]. In the present study, the interaction of targeted miRNAs with either hsa_circ_0067209 or hsa_circ_0000673 was predicted via miRadna and TargetScan softwares, and 13 miRNAs could bind to matched nucleic acid sequences in hsa_circ_0067209 and hsa_circ_0000673 (Figure 6A,B). Subsequently, the expression trends of these candidate miRNAs were measured in PBMC samples from 48 subjects by qRT-PCR assay. By comparing with healthy controls, the results confirmed a marked decrease in expression of hsa-miR-8082 in COPD, which showed an inverse correlation to the expression of hsa_circ_0067209 (Figure 6C,E). In contrast, hsa-miR-1248 expression was dramatically increased in COPD and was negatively associated with hsa_circ_0000673 expression (Figure 6D,F).

**Figure 7:**
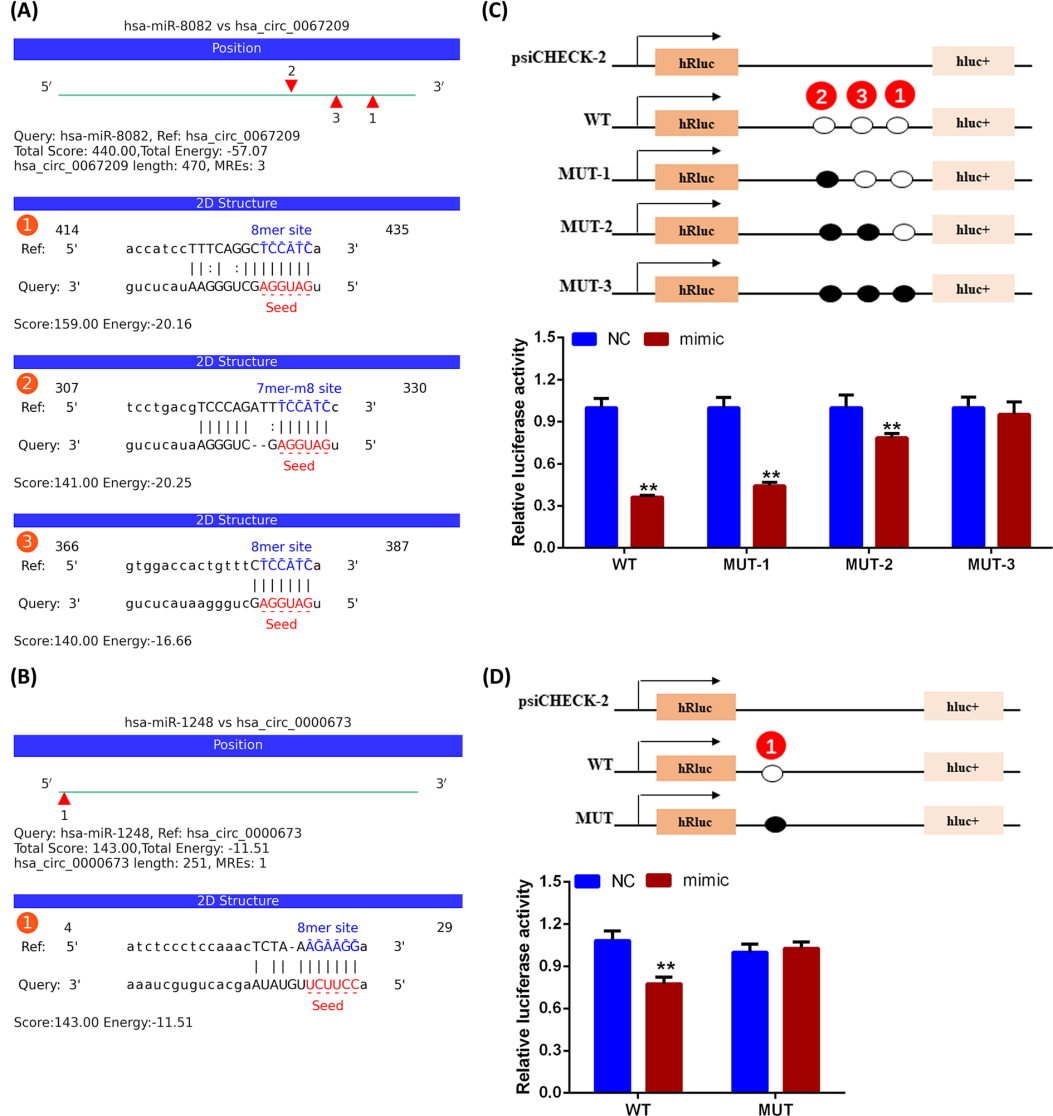
Hsa-miR-8082 and hsa-miR-1248 were direct targets for hsa_circ_0067209 and hsa_circ_0000673, respectively (**A,B**) Potential binding sites were predicted for hsa_circ_0067209/hsa-miR-8082 and hsa_circ_0000673/hsa-miR-1248. (**C,D**) The directed interaction of hsa_circ_0067209/hsa-miR-8082 or hsa_circ_0000673/hsa-miR-1248 was identified by dual-luciferase reporter assay; *P<0.05, **P<0.01.

**Figure 8:**
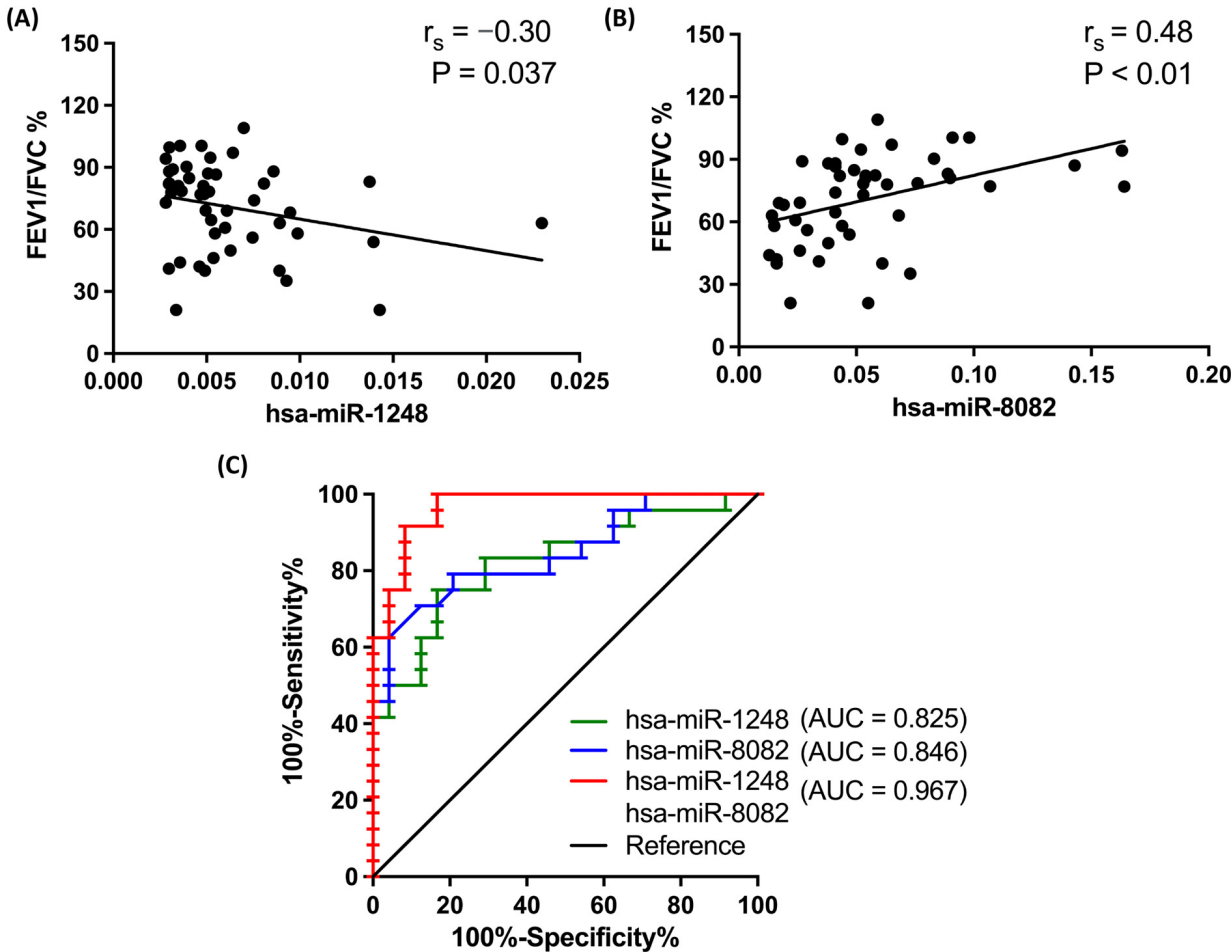
Correlation analysis for clinical characteristics and ROC curve analysis of hsa-miR-1248 and hsa-miR-8082 for COPD (**A,B**) Spearman correlation analysis for the link between FEV1/FVC% and hsa-miR-1248 or hsa-miR-8082 expression. (**C**) ROC curve analysis of hsa-miR-1248 or hsa-miR-8082, and a combination of that two.

The predicted interactions of circRNAs and miRNAs in the present study, as shown in Figure 7A,B, included three binding sites for the interaction of hsa_circ_0067209/hsa-miR-8082, and one binding site for hsa_circ_0000673/hsa-miR-1248. Concurrently, results from the dual-luciferase reporter assay indicated that hsa-miR-8082 and hsa-miR-1248 mimics could significantly decrease the luciferase activity of hsa_circ_0067209 and hsa_circ_0000673, individually, in the WT group, but not in the MUT group (Figure 7C,D). This suggested that there were direct links between hsa-miR-8082 and hsa_circ_0067209, as well as between hsa-miR-1248 and hsa_circ_0000673. In addition, Spearman correlation analysis showed a negative correlation of the expression of hsa-miR-1248 and FEV1/FVC% (Figure 8A). In contrast, hsa-miR-8082 expression was positively correlated with FEV1/FVC% (Figure 8B). The AUC values of hsa-miR-8082 and hsa-miR-1248 were 0.846 (95% CI: 0.736–0.957) and 0.825 (95% CI: 0.705–0.944), respectively, whereas the AUC value of the combination of the two miRNAs was 0.967 (95% CI: 0.924–1.000) (Figure 8C). This suggested that a much better diagnostic value could be provided via the combination of two miRNAs compared with a single-targeted miRNA.

### Construction of circRNA-miRNA-mRNA networks involved in regulation of the COPD process

Since most circRNAs regulate the expression of downstream genes positively through competitive binding with matched miRNAs, the expression profiles of targeted mRNAs were evaluated in three pooled RNA samples per group in PBMCs of COPD patients and healthy controls by RNA sequencing to identify functional circRNA-miRNA-mRNA networks in the present study. Compared with healthy controls, there were 80 DEGs in patients with COPD, based on a threshold (|fold change| >2 and adjusted *P*<0.05), including 44 up-regulated and 36 down-regulated genes (Supplementary Figure S3A–C). The expression changes of all candidate genes are listed in Supplementary Table S2.

**Figure 9:**
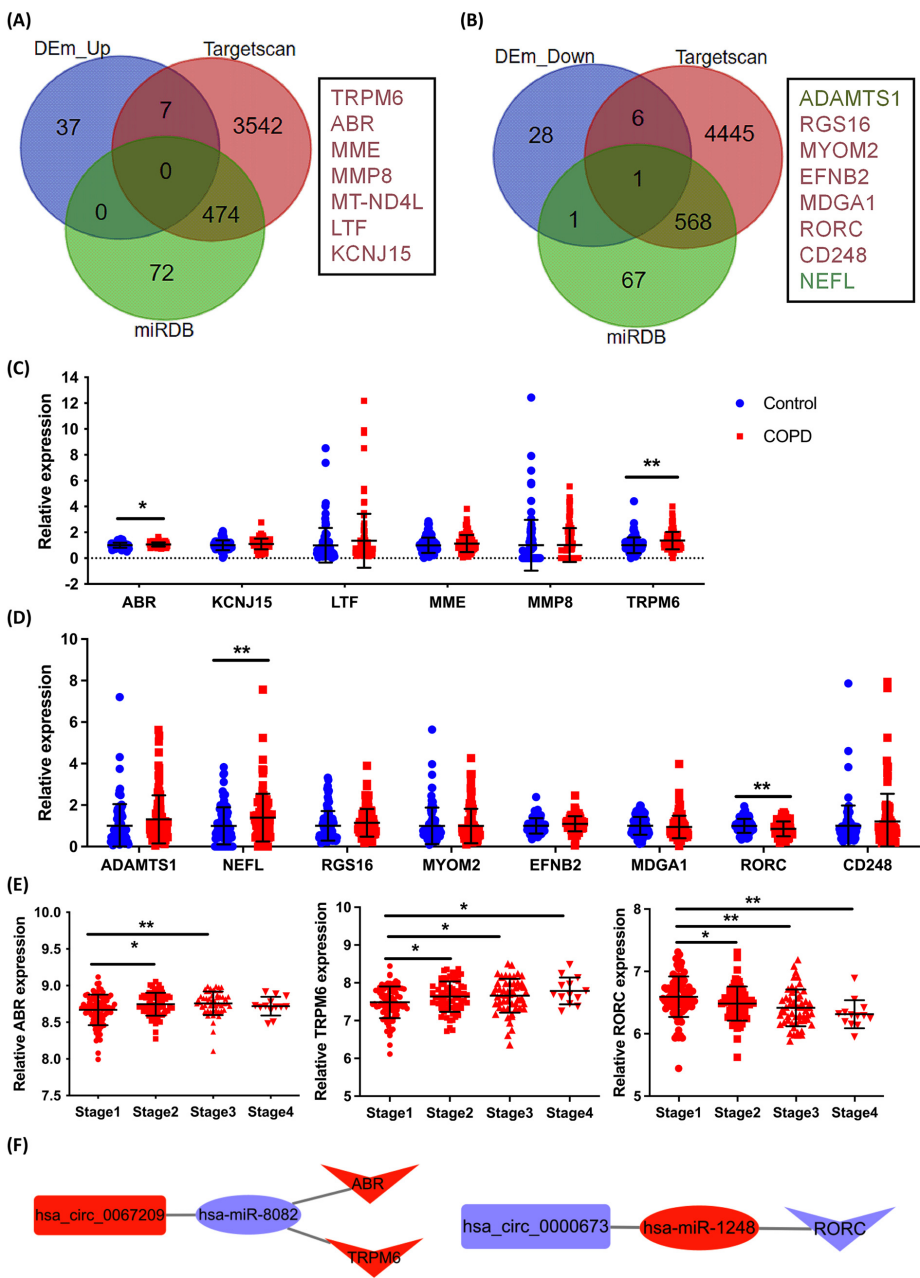
Construction of COPD-related circRNA-miRNA-mRNA networks (**A**) Up-regulated mRNAs predicted to bind to hsa-miR-8082. (**B**) Down-regulated mRNAs predicted to bind to hsa-miR-1248. (**C,D**) Expression levels of targeted genes of hsa-miR-8082 and hsa-miR-1248 in lung tissues of COPD patients and negative controls (COPD: *n*=98, Control: *n*=91). Recalculated datasets were downloaded from the NCBI GEO dataset GSE57148, and the result was presented with probe intensity. (**E**) Expression levels of *ABR*, *TRPM6*, and *RORC* in the blood of patients with COPD were evaluated (Stage 1: *n*=90, Stage 2: *n*=68, Stage 3: *n*=55, Stage 4: *n*=13), according to NCBI GEO dataset GSE54837. (**F**) Construction of the hsa_circ_0067209/hsa-miR-8082/ABR/TRPM6 network and the hsa_circ_0000673/ hsa-miR-1248/RORC network; *P<0.05, **P<0.01.

All 80 COPD-related DEGs were used to construct PPI networks. Consequently, four clusters were generated (see Supplementary Figure S4A). Cluster 1 included seven genes (*MMP8*, *OLFM4*, *BPI*, *CEACAM8*, *CXCL1*, *CAMP*, and *DEFA3*) that are primarily associated with immune response, neutrophil degranulation, and regulation of macrophage activation and are enriched in the NOD-like receptor signaling pathways (Supplementary Figure S4B and S4C). Subsequently, expression profiles of COPD-related DEGs and predicted target genes of hsa-miR-8082 and hsa-miR-1248 were employed to further reveal the molecular functions of circRNA-miRNA-mRNA networks. Overall, seven downstream genes (*TRPM6*, *ABR*, *MME*, *MMP8*, *MT-ND4L*, *LTF*, and *KCNJ15*) corresponded to hsa_circ_0067209/hsa-miR-8082, and eight genes (*ADAMTS1*, *NEFL*, *RGS16*, *MYOM2*, *EFNB2*, *MDGA1*, *RORC*, and *CD248*) were the downstream targets of hsa_circ_0000673/hsa-miR-1248 as interaction networks in the COPD process (Figure 9A,B). Expression profiles of all predicted target genes for hsa-miR-8082 and hsa-miR-1248 were verified by comparing the GSE57148 dataset associated with COPD. This analysis showed that COPD-related *TRPM6* and *ABR* were significantly up-regulated and *RORC* was significantly down-regulated when these gene expressions were compared with those in healthy controls (Figure 9C,D). In addition, these findings were consistent with the negative correlation of miRNAs to corresponding mRNAs.

Ultimately, expression levels of *ABR*, *TRPM6*, and *RORC* were detected in the blood of patients with COPD in the clinical stages of disease (data derived from GEO dataset GSE54837), and based on the severity of disease, mRNA expression levels of *ABR* and *TRPM6* were increased and that of *RORC* was decreased (Figure 9E). The interactive network maps of hsa_circ_0067209/hsa-miR-8082/*ABR*/*TRPM6* and hsa_circ_0000673/hsa-miR-1248/*RORC* in the present study were constructed by Cytoscape software (Figure 9F). Hsa_circ_0067209 and hsa_circ_0000673 may serve as key diagnostic biomarkers for the early diagnosis of COPD and be important regulators of COPD pathogenesis via ceRNA network.

## Discussion

Owing to the high prevalence, morbidity, and mortality, COPD is a serious global threat to human life and health [3,22]. In recent years, numerous studies have focused on screening and identifying novel diagnostic and therapeutic biomarkers of COPD [23–25]. As a special and widespread type of endogenous non-coding RNA, circRNAs are becoming more prominent because they exhibit critical roles in regulating many disease processes. However, there are limited studies on the expression and function of circRNAs in patients with COPD. In the present study, 245 differentially expressed circRNAs, including 111 up-regulated and 134 down-regulated circRNAs, were screened out as COPD-related candidates using microarray analysis. The differentially expressed circRNAs selected for transcriptomics analysis were primarily derived from exons, which was consistent with their distribution in other diseases [26,27]. Validation experiments showed that hsa_circ_0067209 was significantly increased in the PBMC samples of patients with COPD. The targeted host gene of hsa_circ_0067209 is EEFCEC, which is a specialized elongation factor that is crucial in selenoprotein synthesis and may play an important role in maintaining oxidant/antioxidant balance and regulating inflammatory responses [28,29]. Therefore, it is of interest to better understand the roles of hsa_circ_0067209 in the pathogenesis of COPD. A circRNA that was significantly decreased in COPD patients in the present study was hsa_circ_0000673, which comprised the head-to-tail splicing of RSL1D1 exons 4 and 5. A previous study reported that overexpressed hsa_circ_0000673 could act as an oncogene with promising diagnostic value in cholangiocarcinoma [30]. Furthermore, hsa_circ_0000673 could serve as a sponge for miR-767-3p by promoting cell proliferation and invasion in the progression of liver cancer [31]. Collectively, these studies have demonstrated that the expression of hsa_circ_0000673 varies across different diseases.

Based on many reports [14,15,32–34], the effectors of exon circRNAs can serve as miRNA sponges to protect targeted mRNAs from miRNA-mediated degradation. CircRNA-miRNA-mRNA networks have crucial roles in regulating the post-transcription of genes in numerous physiological and pathophysiological processes. To further understand the effects of hsa_circ_0067209 and hsa_circ_0000673 in COPD pathogenesis, based on special axises of circRNA-miRNA and/or miRNA-mRNA were used to construct the ceRNA cross-talk. The interactions of hsa_circ_0067209/hsa-miR-8082 or hsa_circ_0000673/hsa-miR-1248 were subsequently identified by validating the prediction and the regulation of miRNA response elements. Few reports have been published on hsa-miR-8082 research; one clinical trial indicated that hsa-miR-8082 was significantly increased in the prodromal phase of Huntington’s disease [35]. In the present study, hsa-miR-8082 was found to be significantly decreased in PBMCs of patients with COPD, compared with normal controls. Seven DEGs (*TRPM6*, *ABR*, *MME*, *MMP8*, *MT-ND4L*, *LTF*, and *KCNJ15*) were identified as candidate target genes through integration analysis to identify the mRNA expression profiles of COPD. The expression levels of *TRPM6* and *ABR* could be increased due to the severity of COPD, which was consistent with the expectations of the present study. *TRPM6*, a potential transient receptor channel, was previously reported to play a prominent role in regulating vertebrate embryonic development, hypomagnesemia, and metabolic disorders, and may be a promising drug target [36,37]. *ABR*, an activator of RhoGEF and GTPase, is associated with mitosis in human embryonic stem cells, and acts as an apoptotic promoter in dissociated cells [38,39]. Several studies have found that apoptosis of lung structural cells is a factor in the pathogenesis of COPD [40–42]. Recent studies have reported that the dysregulation of hsa-miR-1248 is associated with certain cancers [43–45], diabetes mellitus [46], Sjögren’s syndrome [47], and aging [48], which depends on the roles of inflammatory responses greatly. In the present study, eight mRNAs (*ADAMTS1*, *NEFL*, *RGS16*, *MYOM2*, *EFNB2*, *MDGA1*, *RORC*, and *CD248*) were predicted to be target genes of hsa-miR-1248, with one of them-*RORC*-exhibiting decreased expression with the severity of disease. *RORC* is a key transcription factor for the differentiation of Th17, which can control the expression of several inflammatory genes, and plays key roles in the pathogenesis of COPD [49,50]. Collectively, the results of the present study and previous reports suggest that the hsa_circ_0000673/hsa-miR-1248/RORC axis has a potent effect on the progression of COPD by regulating the inflammatory response.

Clinical data have suggested that a decline in lung function could be accompanied by increased risks of both morbidity and mortality in patients with COPD [51–53]. Therefore, the relationship of lung function and diagnostic value of hsa_circ_0067209/hsa-miR-8082 and/or hsa_circ_0000673/hsa-miR-1248 in COPD were analyzed in the present study. Both hsa_circ_0067209 and hsa_circ_0000673, as well as their miRNAs targets, were notably correlated with FEV1/FVC%. ROC analysis indicated a better AUC value from the combination of hsa_circ_0067209 and hsa_circ_0000673, as well as hsa-miR-8082 and hsa-miR-1248; the AUC values were 0.866 (95% CI: 0.782–0.950) and 0.967 (95% CI: 0.924–1.000), respectively. This finding suggests that the expression levels of the two non-coding RNAs have potential for the clinical application of COPD diagnosis and therapeutics.

Although there are a few novel discoveries revealed by the present study, some limitations remain in our work (such as not big enough sample size, and less verifying tests of biological functions for that two circRNA-associated ceRNA networks). Therefore, we will collect more related-samples of clinical patients and healthy controls, conduct additional follow-up studies to elucidate candidate biomarker profiles, their signal pathways and mechanisms for regulating the COPD process *in vivo* and *in vitro*.

## Conclusions

The present study identifys two novel COPD-related circRNAs, and constructed their own circRNA-associated ceRNA networks (hsa_circ_0067209/hsa-miR-8082/ABR/ and hsa_circ_0000673/hsa-miR-1248/RORC). New findings demonstrate that the expression levels of the two novel circRNAs and their targets might have potential biomarker values for precision diagnosis and therapeutic intervention in patients with COPD.

## Supplementary Material

Supplementary Figures S1-S4 and Tables S1-S2

## Data Availability

The data used to support the findings of this study are available from the corresponding author upon request.

## Abbreviations

CI: confidence interval
circRNA: circular RNA
COPD: chronic obstructive pulmonary disease
DEG: differentially expressed gene
PPI: protein–protein interaction
qRT-PCR: quantitative real-time PCR
ROC: receiver operating characteristic curve
