# Supplementary material for: Identification of circRNA-associated ceRNA networks in peripheral blood mononuclear cells as potential biomarkers for chronic obstructive pulmonary disease
Source: Biosci Rep. 2023 Oct 31;43(10):BSR20230005. doi: 10.1042/BSR20230005 (PMC10619198; doi:10.1042/BSR20230005)
Supplement: Supplementary Figures S1-S4 and Tables S1-S2 [file BSR-2023-0005_supp.pdf]

Supplementary Materials

Supplementary figures

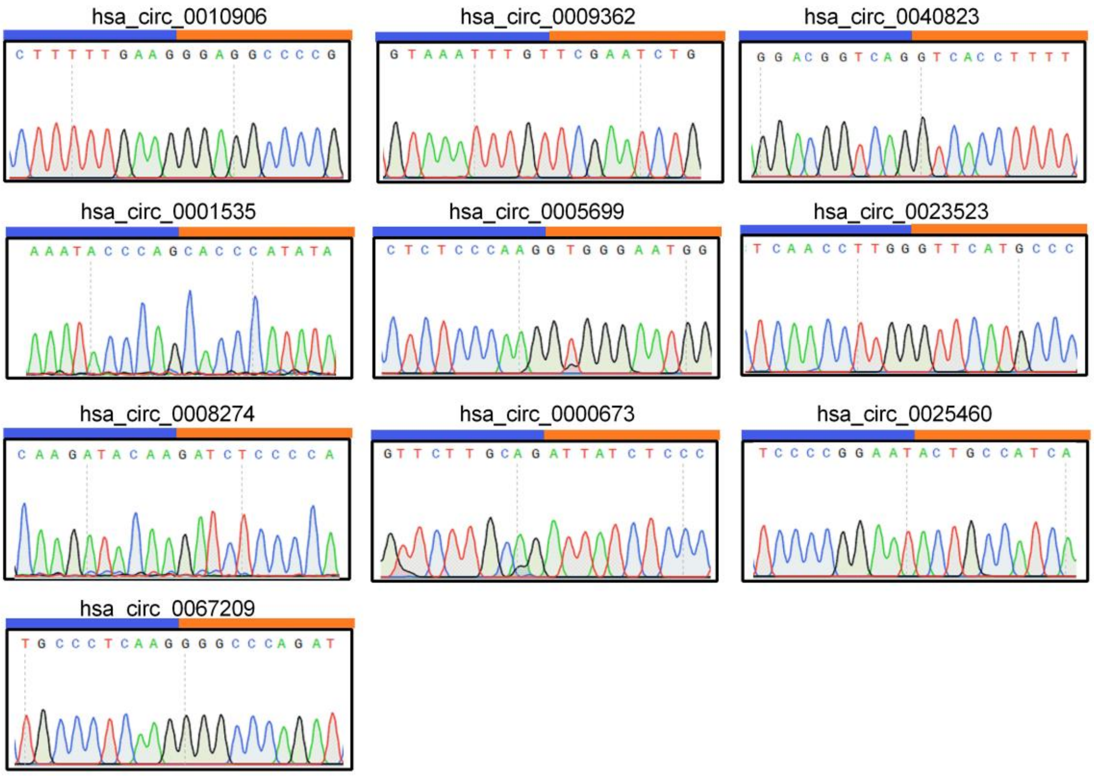

Figure S1 Sanger sequencing of candidate circRNAs related to COPD.

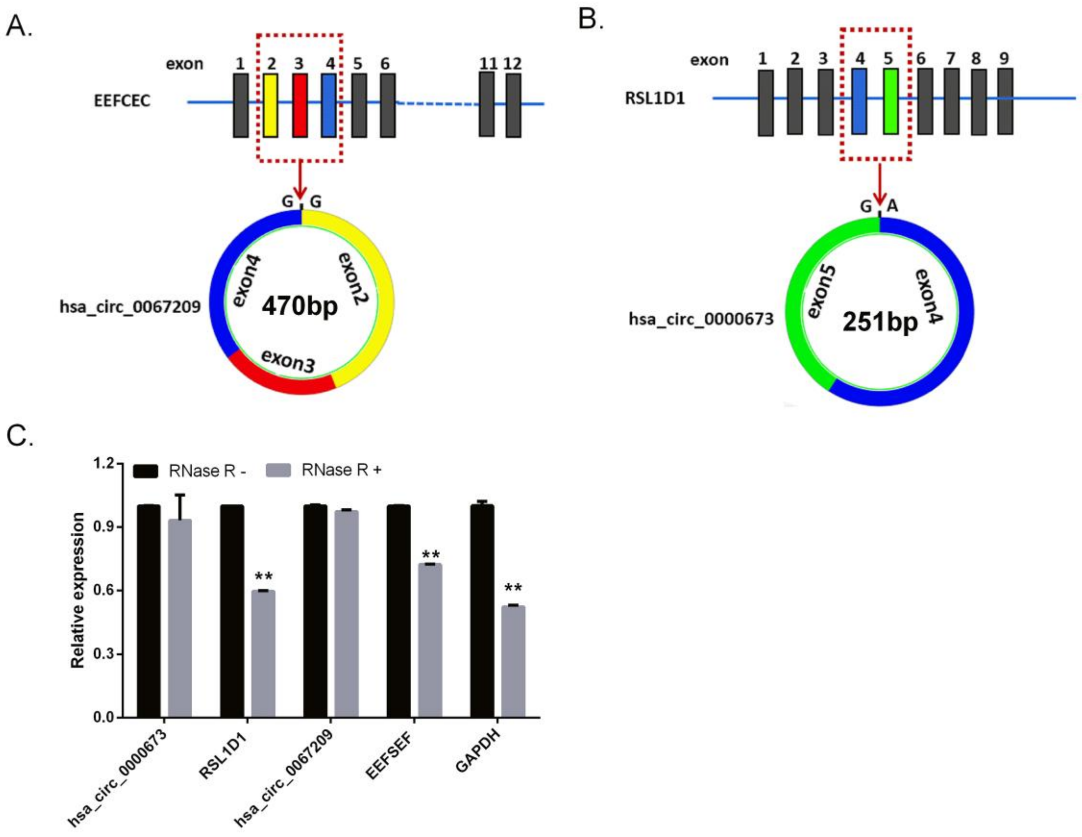

Figure S2 The characteristics of hsa_circ_0067209 and hsa_circ_0000673. A, B) The origin of hsa_circ_0067209 and hsa_circ_0000673. **C)** Expression of hsa_circ_0067209, hsa_circ_0000673, and the host genes were detected by qRT-PCR from Beas-2B cells treated with RNase R (5 U/μg RNA), compared with the RNase R-free group.

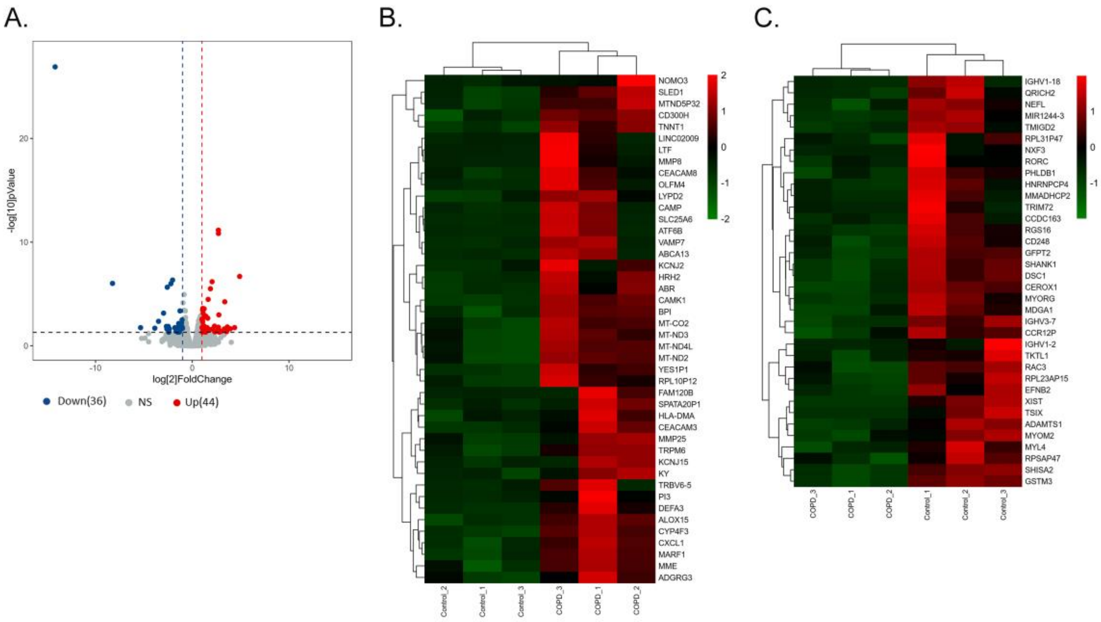

Figure S3 Identification of differentially expressed mRNAs in COPD. A) Volcano map analysis of differentially expressed mRNAs in COPD by RNA sequencing. The red points in the plot represent upregulated mRNAs and the blue points represent downregulated mRNAs, all with fold change >2 and *P* <0.05. **B)** Upregulated and **C)** downregulated mRNAs in the COPD group by hierarchical cluster analysis, compared with the control group. “Red” indicates higher relative expression and “green” indicates lower relative expression.

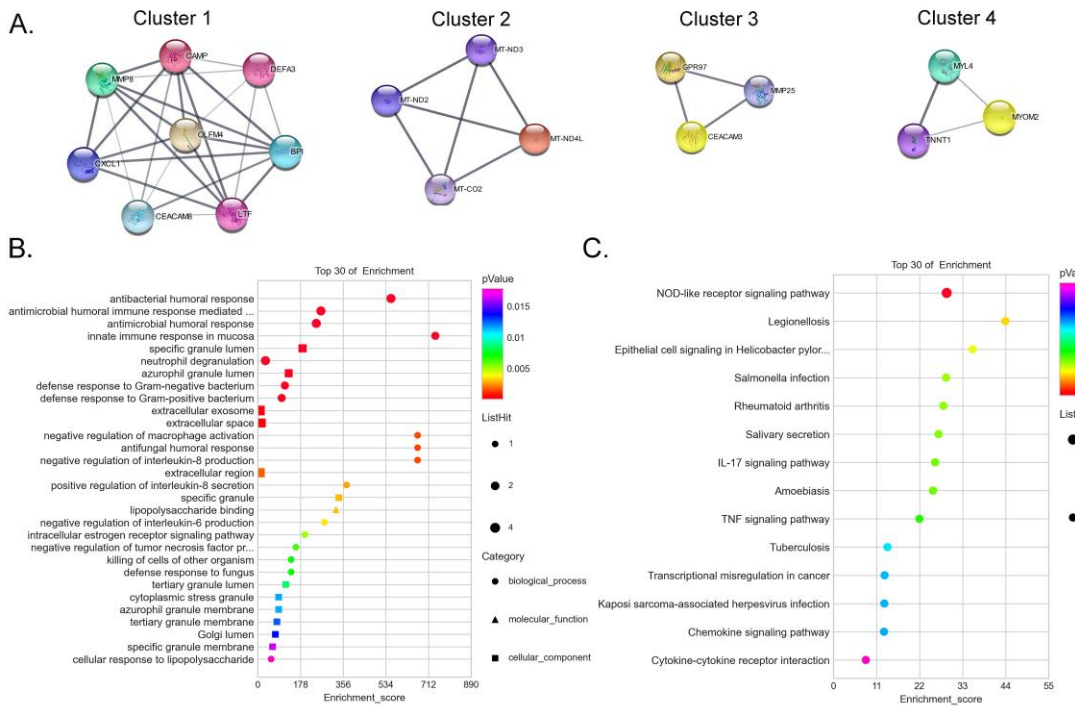

Figure S4 COPD-related protein–protein interaction (PPI) networks and function analysis. A) The clustered modules extracted from COPD-related PPI networks. **B)** GO and **C)** KEGG enrichment analyses of COPD-related genes in most significant modules.

Supplementary tables

Table S1 Primer sequences for validating circRNAs and miRNAs in this study

Gene name	Primer Sequence (5′–3′)	
	**Forward primer**	**Reverse primer**
hsa_circ_0010906	ACAACAAGTTCAAGGGACGC	GGTACACGCTGAAGTTCTGG
hsa_circ_0009362	GTGAGCTTGACCAGTTACGG	GTGCTCTTCAATGCCACCTTC
hsa_circ_0008274	GGGTGGAGTATGATGCTGAGA	ATCACACCAGGTTTCACACCA

hsa_circ_0040823	GAGCAAGTCCAGATCACGCA	TTCTCCGCGAGAAGCTCTTGA
hsa_circ_0023523	GCTCAACCTTGGGTTCATGC	GAGACAAAGCCAGAGGTGATC
hsa_circ_0001535	CAGAGACTGTTCAAAACCTGTG	CATGATCTATGCTGCTCTGAAG
hsa_circ_0005699	CCTGTGTTGCCTACTGCTTCAT	ATGTGGGGCCACTTCCATT
hsa_circ_0025460	AATGTGACTGGCCCGGAT	CAGCTTCTGCACCCTTCTCT
hsa_circ_0000673	AGTGGTTCTTGCAGATTATCTCC	CGCCTAATTCTGGCATCAGTA
hsa_circ_0067209	CACTGTGATGACAGGGACCA	AGCTTCTGGCAGGCAATCTG
EEFSEF	ACTTCAGCCATGCAAGGAGAC	TGGCCCACTGTAATGTGGAAC
RSL1D1	AGAACAGGTTAGAAAGGCAGT	GACCCTCAGTTCTTTACTTGG
GAPDH	CAATGACCCCTTCATTGACC	TTGATTTTGGAGGGATCTCG
hsa-miR-532-3p	CCTCCCACACCCAAGGCTTGCA	
hsa-miR-616	AGTCATTGGAGGGTTTGAGCAG	
hsa-miR-555	AGGGTAAGCTGAACCTCTGAT	
hsa-miR-1303	TTTAGAGACGGGGTCTTGCTCT	
hsa-miR-1243	GGGGAACTGGATCAATTATAGGAGTG	
hsa-miR-1248	ACCTTCTTGTATAAGCACTGTGCTAAA	
hsa-miR-548b-3p	GGCAAGAACCTCAGTTGCTTTTGT	
hsa-miR-767-3p	TCTGCTCATACCCCATGGTTTCT	
hsa-miR-33a-3p	CAATGTTTCCACAGTGCATCAC	
hsa-miR-203a-3p	GTGAAATGTTTAGGACCACTAG	
hsa-miR-25-5p	CATTGCACTTGTCTCGGTCTGA	

hsa-let-7f-2-3p	GGCTATACAGTCTACTGTCTTTCC
hsa-miR-1185-2-3p	CGATATACAGGGGGAGACTCTCAT
hsa-miR-4534	GGATGGAGGAGGGGTCTAAA
hsa-miR-6876-3p	GAGCTGTCTGTGTTTTCCTTCTCAG
hsa-miR-7112-3p	TGCATCACAGCCTTTGGCCCTAG
hsa-miR-488	TTGAAAGGCTATTTCTTGGTC
hsa-miR-519e	AAGTGCCTCCTTTTAGAGTGTT
hsa-miR-874	CTGCCCTGGCCCGAGGGACCGA
hsa-miR-634	AACCAGCACCCCAACTTTGGAC
hsa-miR-1182	GAGGGTCTTGGGAGGGATGTGAC
hsa-miR-324-5p	CGCATCCCCTAGGGCATTGGTG
hsa-miR-515-3p	GAGTGCCTTCTTTTGGAGCGTT
hsa-miR-338-3p	TCCAGCATCAGTGATTTTGTTG
hsa-miR-146b-3p	GCCCTGTGGACTCAGTTCTGGT

Table S2 Differentially expressed mRNAs between COPD patients and healthy controls

Downregulated mRNA			Upregulated mRNA		
**Gene ID**	**Log2FC**	***P*-value**	**Gene ID**	**Log2FC**	***P*-value**
XIST	-14.186	0.000	*MMP8*	4.900	0.000
TSIX	-8.247	0.000	*ATF6B*	4.381	0.018

NXF3	-5.344	0.018	*LTF*	3.938	0.023
IGHV1-2	-3.873	0.020	*SLC25A6*	3.630	0.015
HNRNPCP4	-3.486	0.004	*ABCA13*	3.580	0.023
SHISA2	-2.968	0.001	*SPATA20P1*	3.553	0.043
IGHV1-18	-2.671	0.014	*OLFM4*	3.362	0.000
TRIM72	-2.625	0.024	*TRBV6-5*	3.321	0.024
IGHV3-7	-2.584	0.000	*VAMP7*	3.017	0.039
MMADHCP2	-2.484	0.014	*LINC02009*	2.814	0.049
RPL23AP15	-2.432	0.048	*CAMP*	2.749	0.001
SHANK1	-2.360	0.024	*DEFA3*	2.711	0.000
RGS16	-2.209	0.000	*CYP4F3*	2.702	0.000
ADAMTS1	-2.036	0.000	*PI3*	2.699	0.016
CCDC163	-1.801	0.018	*FAM120B*	2.691	0.021
MYOM2	-1.774	0.020	*NOMO3*	2.470	0.042
RPL31P47	-1.671	0.041	*TRPM6*	2.317	0.044
PHLDB1	-1.582	0.040	*CEACAM8*	2.303	0.013
MIR1244-3	-1.510	0.017	*ALOX15*	2.114	0.023
RAC3	-1.481	0.048	*MARF1*	2.050	0.000
MYL4	-1.452	0.037	*CEACAM3*	1.881	0.000
MYORG	-1.404	0.007	*KY*	1.830	0.028
EFNB2	-1.397	0.021	*TNNT1*	1.643	0.000

CCR12P	-1.395	0.032
CD248	-1.392	0.018
RORC	-1.339	0.047
DSC1	-1.319	0.020
GFPT2	-1.303	0.040
GSTM3	-1.266	0.000
RPSAP47	-1.241	0.027
NEFL	-1.224	0.021
CEROX1	-1.110	0.006
TKTL1	-1.090	0.022
MDGA1	-1.053	0.003
QRICH2	-1.047	0.015
TMIGD2	-1.006	0.021

FC means fold change.

MTND5P32	1.601	0.002
ADGRG3	1.552	0.018
CXCL1	1.402	0.001
MME	1.382	0.048
KCNJ15	1.314	0.047
HLA-DMA	1.301	0.034
MMP25	1.229	0.013
ABR	1.229	0.000
LYPD2	1.179	0.024
SLED1	1.125	0.001
MT-ND2	1.106	0.000
BPI	1.095	0.014
CD300H	1.094	0.002
MT-ND4L	1.088	0.000
CAMK1	1.079	0.005
MT-CO2	1.069	0.000
KCNJ2	1.052	0.048
HRH2	1.043	0.003
RPL10P12	1.030	0.019
YES1P1	1.012	0.018
MT-ND3	1.001	0.003

30

Charlesworth
Author Services

EDITORIAL CERTIFICATE

This document certifies that the manuscript below was edited for correct English language usage, grammar, punctuation and spelling by qualified native English speaking editors at Charlesworth Author Services.

Paper Title:

A study of circRNA-associated ceRNA network in peripheral blood mononuclear cells as potential biomarkers for chronic obstructive pulmonary disease

Author:

Yun Wang

Date certificate issued:

July 26, 2023

cwauthors.com
